# Therapeutic effect against retinal neovascularization in a mouse model of oxygen-induced retinopathy: bone marrow-derived mesenchymal stem cells versus Conbercept

**DOI:** 10.1186/s12886-019-1292-x

**Published:** 2020-01-06

**Authors:** Wei Xu, Weijing Cheng, Xiaoyuan Cui, Guoxing Xu

**Affiliations:** 1 Department of Ophthalmology, The First Affiliated Hospital of Fujian Medical University, 20 Chazhong Road, Fuzhou City, 350005 China; 2 Fujian Institute of Ophthalmology, Fuzhou, China

**Keywords:** Retinal neovascularization, Oxygen-induced retinopathy, Mesenchymal stem cell, Vascular endothelial growth factor, Therapy

## Abstract

**Background:**

To study the therapeutic effect of bone marrow-derived mesenchymal stem cells (BMSC) against retinal neovascularization and to compare with anti-vascular endothelial growth factor (VEGF) therapy.

**Methods:**

Neonatal C57BL/6 mice were exposed in hyperoxygen and returned to room air to develop oxygen-induced retinopathy (OIR). Red fluorescent protein-labeled BMSC and Conbercept were intravitreally injected into OIR mice, respectively. Inhibition of neovascularization and apoptosis in OIR mice were assessed through retinal angiography, histopathology and terminal deoxynucleotidyl transferase-mediated dUTP nick end labeling (TUNEL) assay.

**Results:**

BMSC were able to migrate and integrate into the host retina, significantly inhibit retinal neovascular tufts and remodel the capillary network after injecton. Treatment with BMSC increased the retinal vascular density, decreased the number of acellular capillaries and inhibited retinal cell death. This effect was not inferior to current anti-VEGF therapy by using Conbercept.

**Conclusions:**

Intravitreal injection of BMSC exerts a protective effect against retinal neovascularization and offers a therapeutic strategy for oxygen-induced retinopathy.

## Background

Pathological retinal neovascularization is a major cause of visual diminution and at times even leads to blindness. It refers to the incomplete and unhealthy architecture of the vasculature in many retinal diseases, such as diabetic retinopathy, retinopathy of prematurity and retinal vein occlusion. These diseases involve damage of the retinal vessels, causing exudation of the fluid, hemorrhage or vessel obstruction, and proliferation. This in turn results in retinal ischemia that is associated with severe disorders, such as neovascularization, vitreous hemorrhage and tractional retinal detachment [1].

Anti-vascular endothelial growth factor (VEGF) therapy has led to a breakthrough in the treatment of retinal neovascularization [2]. However, anti-VEGF therapy remains to be controversial in several aspects [3]. For instance, hypertension was discovered when treated with bevacizumab, and lead to other cardiovascular complications [4]. Furthermore, VEGF-A plays an important role as a retinal neuro-protectant, and its blockade under retinal stress conditions accelerates retinal cell death [5]. Emerging research has shown that anti-VEGF-A therapy might be associated with retinal atrophy [6]. Therefore, developing innovative therapeutic strategies against retinal neovascularization is imperative.

Mesenchymal stem cells (MSC) are self-renewing multipotent cells that are presented in the mesenchymal tissues and play important roles in tissue regeneration and injury repair [7]. Recently, important progress has been achieved in understanding the mechanisms of MSC homing and recruitment to the ischemic myocardium [8]. They can be recruited into the neovascularized areas and applied for anti-tumor therapy [9]. MSC could also secrete paracrine factors to promote vascular regeneration [10].

Since MSC are involved in angiogenesis and tissue repair, we decided to investigate the roles of bone marrow-derived mesenchymal stem cells (BMSC) that play during retinal neovascularization in a mouse model of oxygen-induced retinopathy (OIR) to simulate pathogenesis of retinopathy of prematurity. The therapeutic effect of BMSC was compared to Conbercept (Kanghong, Inc., Chengdu, China) which is a fusion protein composed of extracellular domain 2 of VEGF receptor 1 and extracellular domains 3 and 4 of VEGF receptor 2. Intravitreal injection of BMSC, compared to Conbercept, was similarly found to inhibit retinal neovascularization and remodel the capillary network. Besides, BMSCs in our study are proved that it could be able to migrate and integrate into the host retina, which offer a promising treatment strategy for neovascular diseases.

## Methods

### Cell preparation

Commercially available red fluorescent protein-labeled BMSC of C57BL/6 mouse (RFP-BMSC, Catalog No. MUBMX-01201, Cyagen, Guangzhou, China) were cultured to determine the expressions of CD44, CD29, Sca-1, CD31 and CD117, and tested for osteogenic and adipogenic differentiation. The cells were harvested and diluted in Dulbecco’s modified Eagle’s medium (DMEM, Cyagen, Guangzhou, China) at different concentrations for injection (ranged from 5 × 10^6^ cells/ml to 1 × 10^8^ cells/ml).

### Animal experiments

All animal experiments were approved by the animal ethical committee of the Fujian Medical University, and performed in accordance with the National Institutes of Health Guide for the Use of Laboratory Animals. Pregnant C57BL/6 mice (Subline J, Specific pathogen free class) were purchased from Slaccas Animals (Shanghai, China) and housed in room air (21% oxygen) at 25 °C with free access to food and water. After delivery, the neonates together with their mother were exposed to 75% oxygen (hyperoxygen) from postnatal day 7 (P7) to P12 and returned to room air to develop oxygen-induced retinopathy (OIR). Litters at P12 (5.8 ± 0.2 g in weight) were anesthetized and randomly grouped according to the treatments: intravitreal injection of DMEM (1 μl) (OIR-DMEM group), intravitreal injection of RFP-BMSC (1 μl, OIR-BMSC group) and intravitreal injection of Conbercept (1 μl, Kanghong, Inc., Chengdu, China) (OIR-CON group). The litters without hyperoxygen exposure (healthy group) and litters without treatment after hyperoxygen exposure (OIR-blank group) were set as controls.

### Retinal angiography

Mice were anesthetized with an intraperitoneal injection of ketamine (90 mg/kg) and xylazine (8 mg/kg), and administered intraventricular injection of 0.3 ml of FITC-dextran (2000 kDa, 50 mg/ml, Sigma-Aldrich, MO, USA). After 5 minutes, the mice were euthanized by intraperitoneal injection of sodium pentobarbital (200 mg/kg) and the eyes were enucleated and then fixed with 4% paraformaldehyde for 2 h. The corneas and the lenses were removed under a stereo microscope (66 VisionTech, Suzhou, China). The retinas were carefully dissected and flat-mounted on a glass slide with anti-fluorescent quenching solution. The retinal vessels were viewed by fluorescence microscopy (Zeiss Axiophot, NY, USA). The avascular areas and neovascular tuft areas were assessed to evaluate the aspects of angiogenesis and treatment outcomes. The retinas were digested in 3% trypsin (Gibco, CA, USA) for 2–3 h at 37 °C to isolate the retinal vasculature. Subsequently, the retinal vasculature was stained with periodic acid–Schiff reagent and hematoxylin to investigate the capillary network.

### Retinal histopathology and TUNEL assay

Mice were euthanized by intraperitoneal injection of sodium pentobarbital (200 mg/kg) and eyes were enucleated and immediately placed in 4% paraformaldehyde for 24 h, dehydrated using a graded ethanol series, and embedded in paraffin. Sagittal sections were cut from each eye. Serial sections of 5 μm thickness were cut, deparaffinized in xylene and then hydrated. Hematoxylin and eosin staining was used to quantify neovascularization by counting the vascular nuclei that are extended anteriorly from the internal limiting membrane into the vitreous. The average number of neovascular nuclei per section was calculated in each group. The retinal vascular nuclei were counted using a masked protocol by light microscopy (magnification, 100–250). A terminal deoxynucleotidyl transferase-mediated dUTP nick end labeling (TUNEL) assay kit was used to test apoptosis in the retinal sections according to the manufacturer’s instructions (Roche, IN, USA). Images were captured under a Zeiss fluorescence microscope. The number of TUNEL-positive cells was calculated by Image-Pro plus 6.0. Images from random quadrant of posterior retinas were selected and masked to investigators for statistical analysis.

### Statistical analysis

Each experiment was replicated three times. The normally distributed data are presented as mean ± standard deviation (SD). One-way ANOVA was used for comparison among groups in each independent experiment followed by LSD test as post-hoc analysis. Statistical significance was set at *P* < 0.05.

## Results

### Evaluation of retinal neovascularization

**Fig. 1:**
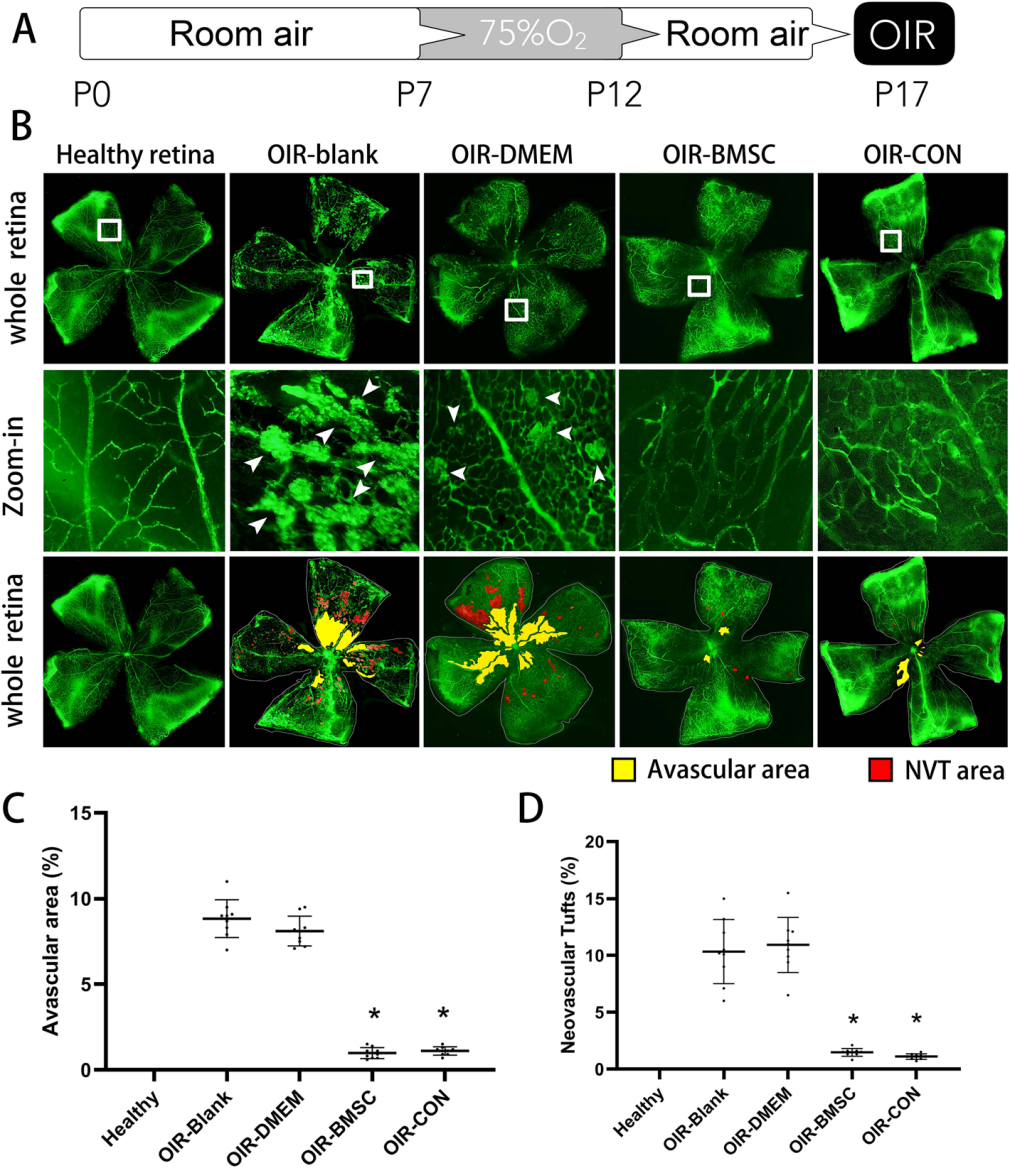
Neovascularization was inhibited after BMSC or Conbercept injection. **a** Schematic process of establishing an OIR animal model by exposure to 75% oxygen from postnatal day 7 (P7) to P12 and return to room air with or without intravitreal injection. **b** P17 retinal flat-mount revealed neovascular tufts (NVT, arrowheads) and avascular areas developed after high oxygen exposure (OIR-blank and OIR-DMEN) but shrinked following BMSC (OIR-BMSC) or Conbercept (OIR-CON) injection. **c** Avascular areas were presented as percentages to the whole retina; both BMSC and Conbercept injection significantly reduced avascular areas. **d** Similar quantification was calculated in regard to the neovascular tufts (NVT) areas. *, *P* < 0.05 versus OIR-blank; *n* = 9

After hyperoxygen exposure from postnatal day 7 (P7) to P12, the neonatal mice developed OIR in the room air. OIR reached a peak severity at P17 during observation. The retinas were flat-mounted and examined at P17 to evaluate the vascular morphology in different groups. The superficial and deep vascular layers that are extended from the optic nerve to the periphery were observed in the retinas of healthy group, which showed the existence of fewer neovascular complexes. In contrast, multiple neovascular tufts and central avascular areas were observed in the retinas of OIR-blank and OIR-DMEM groups (red and yellow zone, Fig. 1b). The retinas from hyperoxygen-exposed mice treated with either BMSC or Conbercept showed fine radial branching pattern in the vessels and significantly reduced the neovascular tufts and the avascular areas. The avascular areas and the neovascular tufts in OIR-BMSC and OIR-CON groups were significantly smaller than those in OIR-blank and OIR-DMEM groups (*P* < 0.05), while no significant difference existed between OIR-BMSC group and OIR-CON group (Fig. 1c, d).

**Fig. 2:**
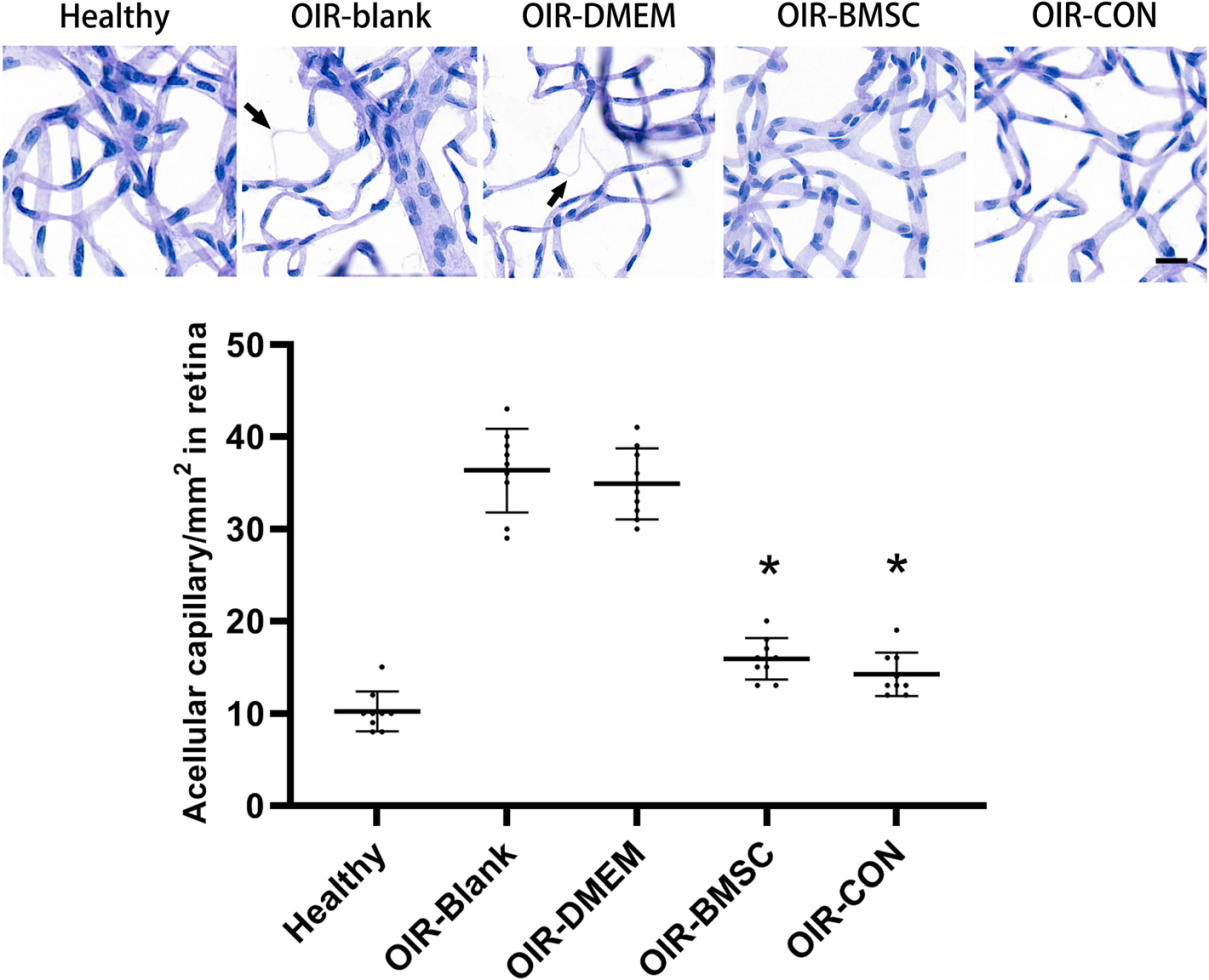
Retinal vascular density and acellular capillaries among groups. Retinal flat-mount digestion showed vascular density was improved after BMSC or Conbercept injection. Acellular capillaries (arrows) appeared in OIR-blank and OIR-DMEM but reduced in OIR-BMSC and OIR-CON. Quantification revealed significant improvement in the groups receiving BMSC or Conbercept injection (P17). *, *P* < 0.05 versus OIR-blank; *n* = 9; Bar = 20 μm

In the whole mount retinal digests, the endothelial nuclei and pericytes nuclei were elongated and spherical, respectively. The endothelial nuclei decreased in the capillaries of OIR-blank group and OIR-DMEM group, as observed that fusiform nuclei relatively increased in OIR-blank and OIR-DMEM groups. Acellular capillaries were calculated per mm^2^. Compared with hyperoxygen exposure without treatment, both BMSC and Conbercept treatment significantly reduced the number of acellular capillaries in the retinas (*P* < 0.05, Fig. 2). Further comparison revealed no significant difference between BMSC and Conbercept treatment (*P*>0.05).

### BMSC recruitment after intravitreal injection

**Fig. 3:**
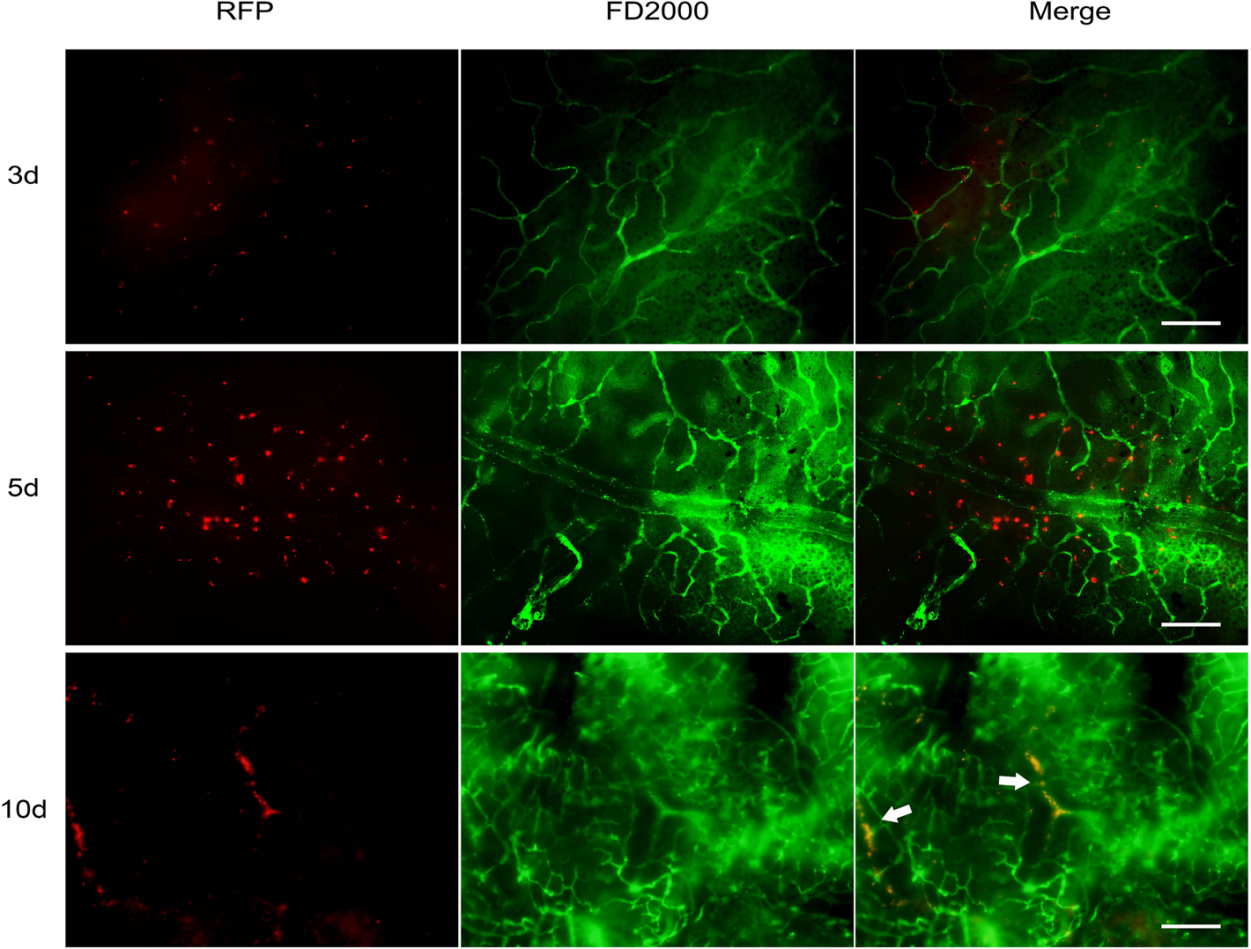
Recruitment of RFP-BMSC to retinal vessel. RFP-BMSC were specifically recruited to the inner surface of retina 3 days after injection. The cell density increased at day 5. Recruited cells integrated into the retinal vasculature and displayed dendritic appearance 10 days after injection. Bar = 200 μm

To examine whether BMSC was specifically recruited to the sites of retinal neovascularization, we detected the expression of red fluorescent protein (RFP) in the retinas of 3 days (P15), 5 days (P17) and 10 days (P22) after BMSC injection. The results showed few RFP-positive cells in the retinal vascular areas at P15. The cells were increased and dispersed around the retinal vascular sites at P17, indicating that the BMSC migrate closer to the vessels. Retinal flat-mount at P22 revealed that the cells participated in the formation of vascular structure (Fig. 3).

### Retinal histology and apoptosis assay

**Fig. 4:**
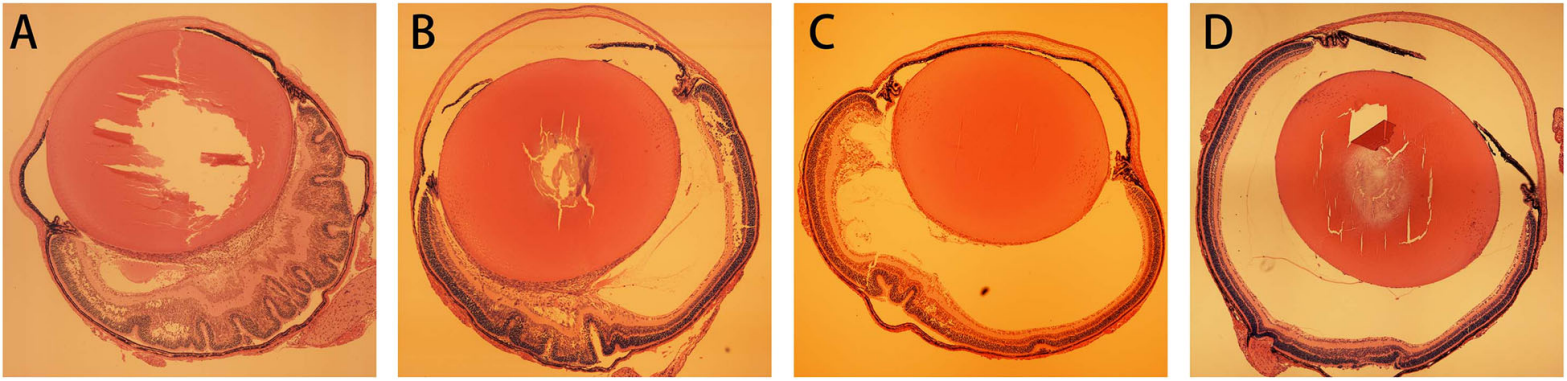
Vitreoretinal appearance after BMSC injection by a series of concentrations. **a** Cell concentration at 1 × 10^8^ cells/ml resulted in severe retinal traction and proliferation in the vitreous. **b** Traction and proliferation reduced when the cell concentration was adjusted to 5 × 10^7^ cells/ml. **c** Vitreoretinal appearance was improved at 1 × 10^7^ cells/ml, but mild traction and proliferation still existed. **d** Traction and proliferation disappeared when tapering to 5 × 10^6^ cells/ml

**Fig. 5:**
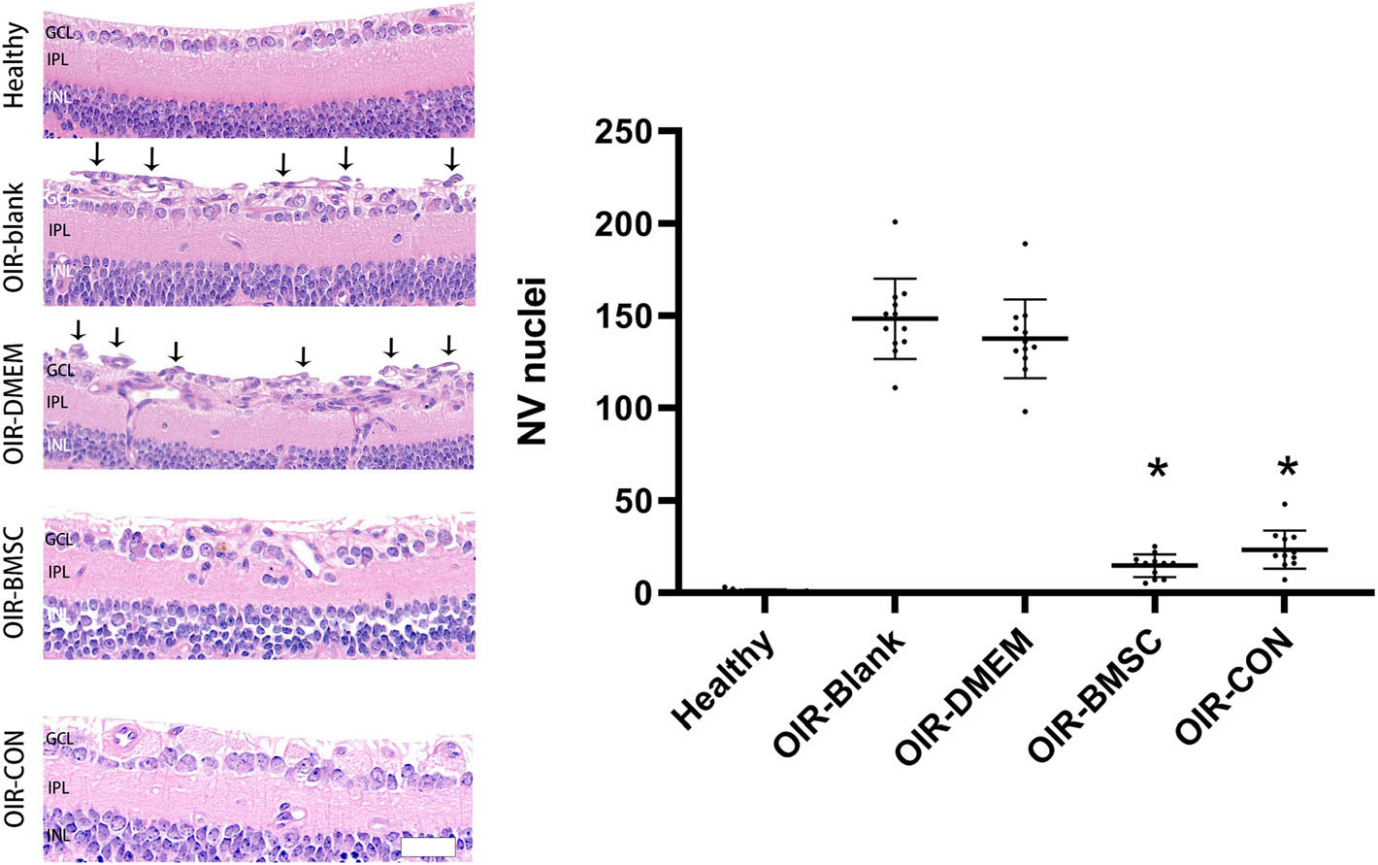
Histological analysis of neovascularization (NV) among groups. Abnormal NV extended beyond the inner limiting membrane in OIR-blank group and OIR-DMEM group, forming lumen-like structures (arrows), while the inner surface of healthy retina appeared clear (P17). Neovascular nuclei in OIR-BMSC group and OIR-CON group significantly reduced in contrast to OIR-blank by random field counting in posterior retina, while no significance existed between OIR-BMSC group and OIR-CON group. *, *P* < 0.05 versus OIR-blank; *n* = 12; Bar = 50 μm

The severity of proliferation in the vitreous after injection is proportional to the concentration of BMSCs (Fig. 4). Therapeutic dose was thereby tapered to 5 × 10^6^ cells/ml. The extent of neovascularization was quantified in the retinal sections by counting the average number of vascular nuclei that extend beyond the inner limiting membrane into the vitreous body. There were no neovascular nuclei in the healthy group. Both OIR-blank group and OIR-DMEM group showed active neovascularization, and are characterized by lumen-like structure accompanied with nuclei located above the inner limiting membrane. In contrast, fewer blood vessels were observed beyond the inner limiting membrane in OIR-BMSC group and OIR-CON group (Fig. 5). Neovascular nuclei in OIR-BMSC group and OIR-CON group were significantly lower than those in OIR-blank group (*P* < 0.05). Intravitreal injection of either BMSC or Conbercept significantly attenuated the neovascularization response with no difference between BMSC and Conbercept (*P*>0.05).

**Fig. 6:**
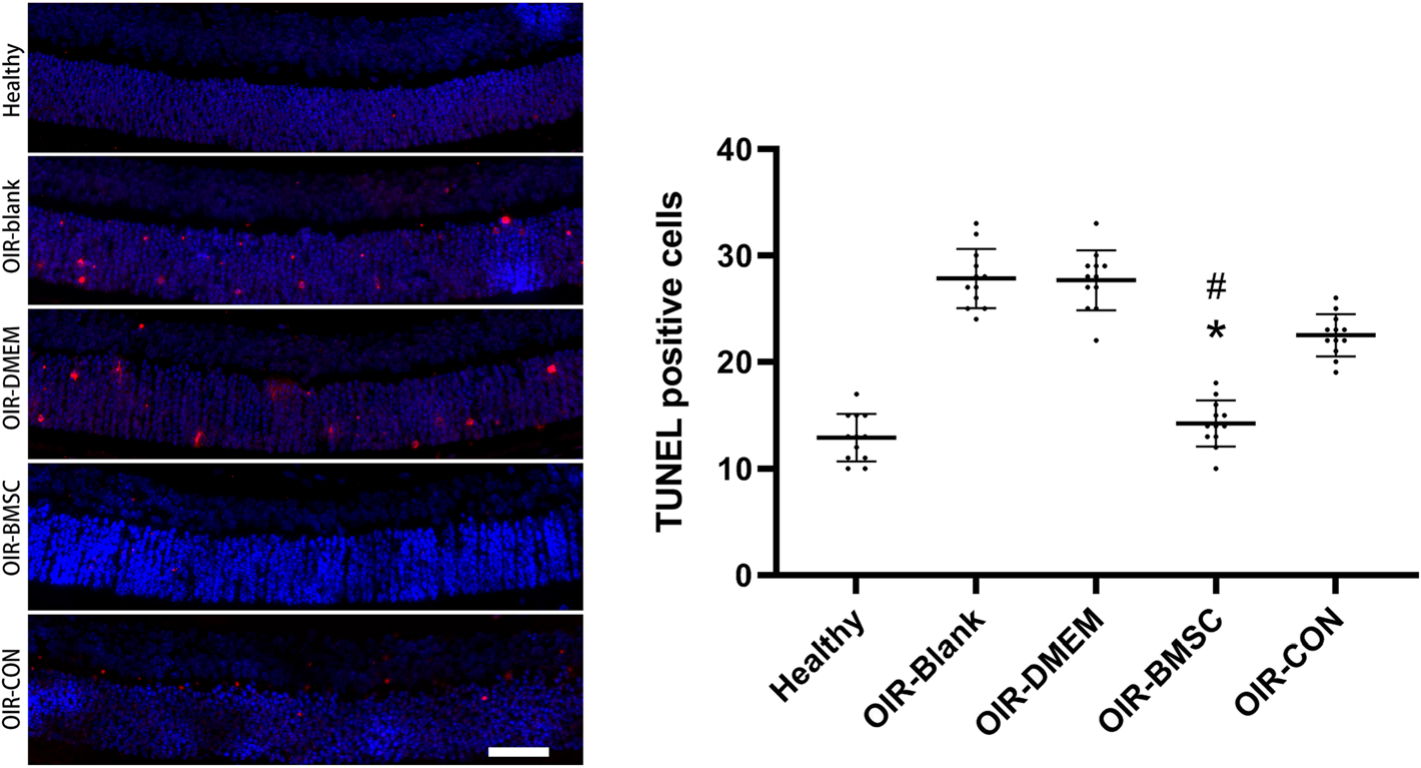
BMSC injection inhibited apoptosis in the retinal cells. Apoptosis mainly occurred in the retinas of OIR-blank group, OIR-DMEM group and OIR-CON group, while fewer apoptotic cells appeared in OIR-BMSC group or healthy group. Quantification of TUNEL-positive cells among groups by random field counting in posterior retina revealed significant inhibition of apoptosis in the group receiving BMSC in contrast to OIR-Blank and OIR-CON (P17). *, *P* < 0.05 versus OIR-Blank; #, *P* < 0.05 versus OIR-CON; *n* = 12; Bar = 50 μm

We also performed the TUNEL assay to determine the apoptotic response in the retina during vascular tuft regression. BMSC injection significantly reduced the number of TUNEL-positive cells in contrast to OIR-blank group (*P* < 0.05), while Conbercept injection showed no significant difference (Fig. 6). Further comparison in regard to the apoptosis of retinal cell between BMSC and Conbercept treatment also revealed significant difference (*P* < 0.05). This suggested that the BMSC have anti-apoptotic effects in hypoxic environment.

## Discussion

Several previous studies have provided evidence regarding the role of BMSC in ocular neovascularization. In this study, we found that the BMSC remodeled the vascular network and reduced the avascular areas and neovascularization. Similar effects were observed in multiple animal models of retinal neovascularization by using human BMSC [11]. However, we specifically examined the role of BMSC and compared with Conbercept, which is an important anti-VEGF drug that is clinically used for the treatment of ocular neovascularization. Although higher doses of intravitreal injection of BMSC tend to induce proliferative retinopathy, our results revealed that lower dose injection of BMSC yielded protective effects against retinal neovascularization, but not inferior to anti-VEGF therapy.

The putative retinal neovascularization tropism is primarily based on the innate physiological ability of BMSC to move to the sites of inflammation and repair the tissues [12]. Retinal neovascularization is a component of several key processes associated with ischemic retinopathies [13]. Hyperoxygen-exposure followed by room air at P12 resulted in retinal hypoxia, leading to elevated levels of hypoxia-related cytokines that contribute to the formation of avascular areas and retinal neovascularization. These cytokines also contribute to the differentiation of BMSC into endothelial-like cells [14], which is partly the reason as to why BMSC injection exerted therapeutic effects. Apart from angiographic improvement by BMSC, significantly reduced retinal cell death was also observed in the OIR mice. Although no trophic factors were detected in this study, our previous report suggested that the trophic or paracrine effect of BMSC may promote this tissue repair [15].

Specific recruitment to the location of ischemic area is required for BMSC to participate in tissue repair. The interaction between stromal cell-derived factor-1 (SDF-1) and CXC-motif-chemokine receptor 4 (CXCR4) is generally considered to be essential for the recruitment of BMSC during injury. Our previous study reported that the stromal cell-derived factor 1a (SDF-1a) stimulated MSC elicited superior effects in terms of both MSC migration and inhibition of apoptosis [16]. But recently, the hypothesis that VEGF and SDF-1 promotes the recruitment of bone marrow-derived cells in retinal neovascularization is gaining acceptance [17]. Therefore, further investigation into BMSC recruitment during retinal neovascularization is necessary to understand how these cells participate in vascular remodeling.

Retinal neovascularization is tightly regulated by a dynamic and natural equilibrium between local proangiogenic and antiangiogenic factors [1, 18, 19]. Among these factors, VEGF and PEDF are the major components, and retinal neovascularization involves disequilibrium of VEGF/PEDF. Rebalance of these angiogenic stimulators and inhibitors might play a crucial role in attenuating retinal damage [20]. Whether BMSC play a role in equilibrium between VEGF and PEDF requires further investigations. Interestingly, there was significant difference in the TUNEL assay where the BMSC injection showed better performance. Also, further investigations into the functional recovery are required to determine whether BMSC injection yields better visual outcomes.

Pathological angiogenesis is a hallmark in the pathogenesis of retinopathy of prematurity, and is most evident during the hypoxic phase following hyperoxic exposure [21]. The animal model of OIR in this study simulated the hyperoxic and hypoxic phase in the retinopathy of prematurity though oxygen control. Although our findings provide a therapeutic strategy against retinopathy of prematurity, attention should be paid on the induction of potential proliferative vitroretinopathy with BMSC injection. Still we were able to provide the therapeutic effect by tapering the injection dose. Safety is the most important concern in the application of BMSC against retinal neovascularization. Several clinical trials regarding the safety of these cells in retinal disorders have been put forwarded and showed satisfactory results [22–24]. Therefore, the application of BMSC in retinal neovascularization is highly anticipated despite these limitations.

## Conclusions

Our study suggested that BMSC inhibit neovascularization and exert a protective effect on OIR. This protective effect is associated with the recruitment of BMSC to the site of lesion and reduction in the retinal cell apoptosis. Our study provides an alternative therapeutic strategy to inhibit neovascularization in neovascular disease by BMSC injection instead of current anti-VEGF therapy.

## Data Availability

The datasets used and analysed during the current study are available from the corresponding author on reasonable request.

## Abbreviations

BMSC: Bone marrow-derived mesenchymal stem cells
OIR: Oxygen-induced retinopathy
VEGF: Vascular endothelial growth factor
